# Foam sclerotherapy for patients with Klipple-Trenaunary syndrome complicated by frequent cellulitis of lower extremity: A case report

**DOI:** 10.1097/MD.0000000000036011

**Published:** 2023-11-17

**Authors:** Fandong Li, Mengtao Wu, Peng Wu, Dianjun Tang

**Affiliations:** a Department of Vascular Surgery, The Second Hospital, Cheeloo College of Medicine, Shandong University, Jinan, China.

**Keywords:** cellulitis, foam sclerotherapy, Klipple-Trenaunary syndrome, lower extremity, lymphedema, venous hypertension

## Abstract

**Rationale:**

Klipple-Trenaunary Syndrome (KTS) complicated by frequent cellulitis of lower extremity seriously affects a patient quality of life. The hemodynamic characteristics of the disease are still unclear. Direct skin incision or puncture to remove malformed veins at the lesion site carries the risk of non-healing of the surgical incision. Our aim is to explore initial management strategies based on the hemodynamic characteristics of this disease.

**Patient concerns:**

A 29-year-old Manchu man was affected by KTS from childhood, characterized by an increase of the circumference and superficial varicose veins of the lower extremity. In the past 5 years, he suffered from frequent cellulitis in the left leg every 15 days or so.

**Diagnoses:**

KTS complicated by frequent cellulitis of lower extremity.

**Interventions:**

The clinical and hemodynamic characteristics of KTS were evaluated by Doppler ultrasonography (DUS) combined with CT venography (CTV), and foam sclerotherapy and postoperative elastic bandage compression were performed accordingly.

**Outcomes:**

Based on evaluations, the reason for frequent cellulitis was the continuous increase of venous hypertension in the calf caused by the malformed superficial vein and its penetrating vein. After 3 operations, the patient had no recurrence of cellulitis of the leg. Follow-up for 1 year showed no recurrence of left leg cellulitis.

**Lessons:**

This report emphasizes that foam sclerotherapy can significantly improve the clinical symptoms of KTS, such as cellulitis, and provide a safe skin environment for the implementation of other surgical methods, based on the evaluation of the pathological characteristics of KTS by DUS combined with CTV.

## 1. Introduction

Klippel-Trenaunay syndrome (KTS) is a rare congenital disorder that usually affects the capillary, venous, and lymphatic systems of the lower extremities.^[1,2]^ The acute phase of frequent cellulitis complicated with KTS is characterized by redness, swelling and pain of the lower leg accompanied by high fever, which seriously affects the patient quality of life.^[3]^ Until now, the etiology and hemodynamic characteristics of the disease have been unclear.^[4]^ Treatment of the disease also aims to improve the patient quality of life.^[5,6]^ Surgical, endovascular laser or radiofrequency ablation of superficial varicose veins or venous lymphatic anastomosis have been reported to reduce lymphedema, but there is a risk of nonunion of the surgical incision.^[7–9]^ In our case, Doppler ultrasonography (DUS)combined with CT venography (CTV) was used to evaluate the clinical and hemodynamic characteristics of KTS, and foam sclerotherapy was applied according to the characteristics of KTS, which successfully prevented frequent cellulitis in the patient with KTS and significantly improved his quality of life.

## 2. Case presentation

A 29-year-old Manchu man was referred to our hospital due to frequent cellulitis in the left leg for 5 years. He was affected by KTS from childhood, characterized by an increase of the circumference and superficial varicose veins of the lower extremity. In the past 5 years, he suffered from frequent cellulitis in the left leg every 15 days or so. In the onset of acute cellulitis, the clinical manifestations were skin redness, high skin temperature, pain, and the highest body temperature of 39.8°C, which seriously affected his quality of life. Elastic compression treatment with elastic bandage was performed intermittently at another hospital, but these symptoms still did not improve and our hospital was subsequently consulted. No family history of KTS was recorded. He has smoked 12 cigarettes a day for 10 years.

**Figure 1.**
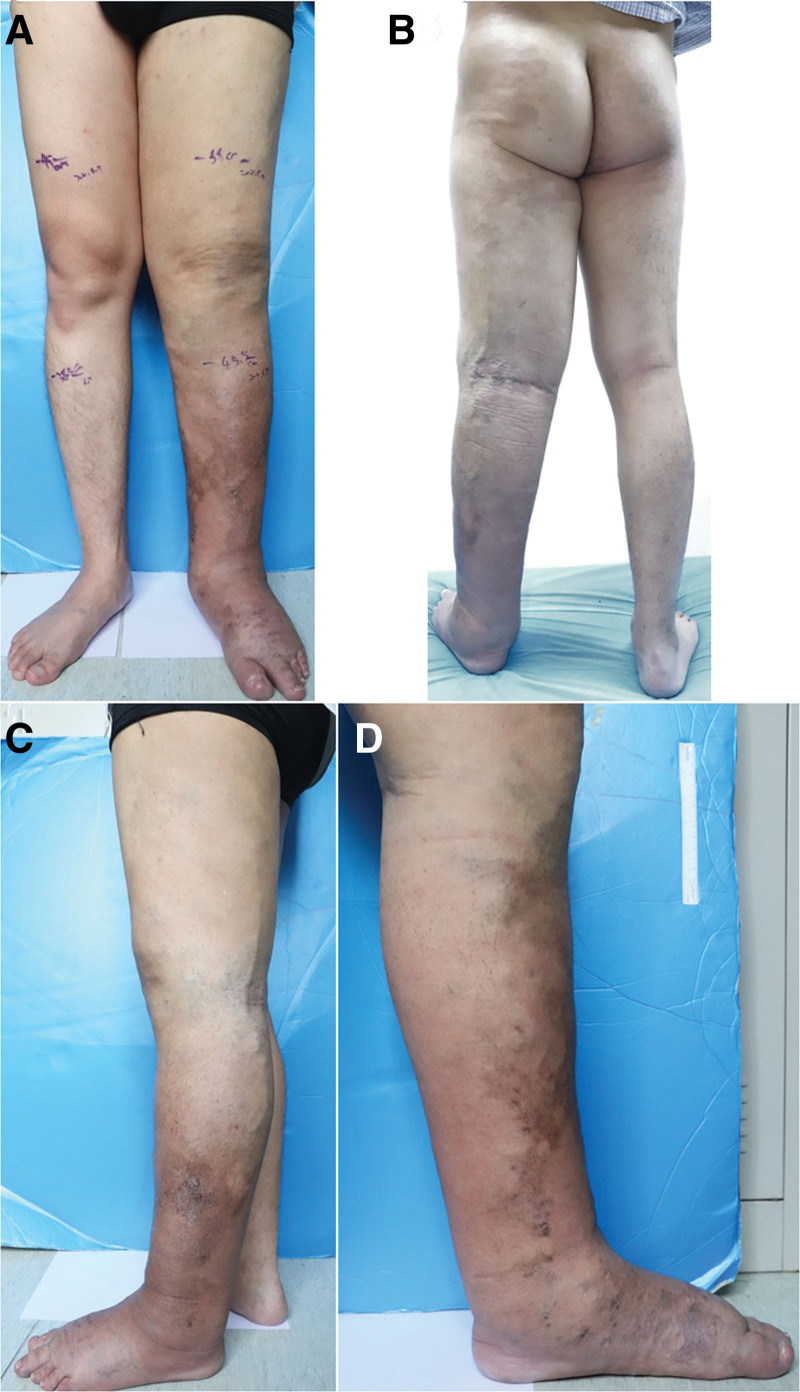
Lower extremity appearance at admission in a man with Klipple-Trenaunary Syndrome. (A) Left full leg swelling, including Buffalo - Hump foot Dorsum and square shape toes. (B) A posterior view reveals brown pigmentation spots on the lateral buttocks and femoral and posterolateral varicose veins of the lower extremities. (C and D) The skin on the anteromedial and lateral sides of the left tibia showed brown pigmented spots, and the rest was dark red.

At admission, his body temperature was 36.2°C. Swelling was detected in the whole left lower extremity, including buffalo-hump foot dorsum and square-shaped toes (Stemmer sign). The left foot was warm with palpable pulses. Visible varicosities were observed throughout the whole left leg and the port-wine birthmark was on the lateral buttocks and posterolateral thighs. The skin on the anteromedial and lateral sides of the left tibia showed brown pigmented spots, and the rest was dark red with high skin temperature and poor elasticity. There was no discrepancy in leg length. The circumference (cm) of both lower extremities was measured as follows:10 cm below the knee joint, 45.5 (left), 38.5 (right); and 10 cm above the knee joint, 58 (left), 49 (right). The right lower extremity was normal (Fig. 1). Laboratory tests showed normal routine blood count, coagulation parameters, and liver and kidney function parameters.

DUS showed the small diameter of superficial femoral vein (2.6 mm) and the popliteal vein (3.0 mm) in the left lower extremity, good intraluminal sound permeability and complete blood flow signal; an anatomically malformed superficial vein (11.9 mm in diameter) in the superficial fascia of the left lower extremity with missing vein valve, rising from the dorsum pedis, passing through the lateral side of the calf and, the lower and middle thigh, the anteromedial side of the upper thigh, and finally draining into the root of the great saphenous vein; several perforating veins on the lateral side of the calf (about 9.7 mm in the maximum diameter), passing between the deep vein and the malformed superficial vein. Squash the proximal extremity and the bidirectional color Doppler blood flow signal was shown in the perforating vein.

**Figure 2.**
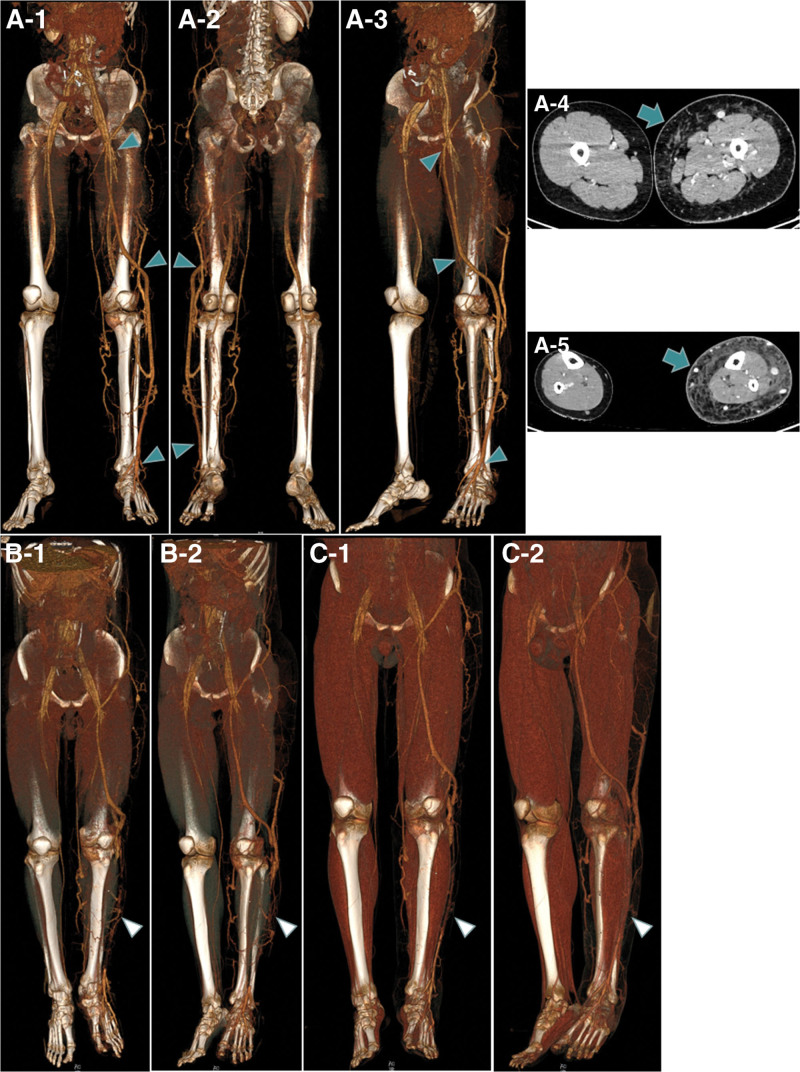
Lower extremity CT angiography in a man with Klipple-Trenaunary Syndrome at admission and at 3 and 6 mo postoperatively. A-1, A-2, and A-3, CT volume imaging of left lower extremity vein distribution at admission. A-4 and A-5, Axial CT image of superficial lower extremity fascia at admission. B-1 and B-2, CT images at 3 mo postoperatively revealed the malformed vein occlusion on the lateral side of the left calf and the open perforating vein. C-1 and C-2, CT images at 6 mo postoperatively showed partial recanalization of the distal malformed vein which had been occluded on the lateral left leg, and occlusion of the perforating vein.

CT volume imaging visually showed what was seen on ultrasound. The axial CT image showed lymphedema within the superficial fascia (Fig. 2A).

According to the above data, the reason for frequent cellulitis was the continuous increase of venous hypertension in the calf caused by the malformed superficial vein and its penetrating vein, which affected lymphatic circulation at the same time. Removing venous hypertension from the malformed venous system might relieve the patient symptoms of clinical discomfort. Considering that the patient lower leg skin was not healthy and that the incision might increase the chance of cellulitis, we opted for foam sclerotherapy.

A total of 3 operations were performed, each with an interval of 3 months.

In first operation, through the right femoral vein puncture approach, the catheter was superselected to enter the malformed vein of the left leg, and 6 ml foam agent (3% polypolycarol injection 2 mL + air 3 mL) was injected. Eccentric compression of the treated malformed venous segment was performed immediately after injection and continued for 1 week. Three months later, the patient had no recurrence of cellulitis of the leg. CT showed that the perforating vein associated with the malformed vein had not yet closed (Fig. 2B). Therefore, foam sclerotherapy (1% polypolycarol injection 2 mL + air 3 mL) was performed on the perforating vein under ultrasound guidance during the second operation. Six months later, the patient reported no recurrence of cellulitis after a long trip. CT showed that the perforating vein was closed, but the malformed vein that had been closed above the lateral malleolus was partially recanalized (Fig. 2C). In the third operation, foam sclerotherapy for superficial varicose veins of the lower extremities was also performed under ultrasound guidance. The following 2 postoperative eccentric compression methods were the same as the first. Follow-up for 1 year showed no recurrence of left leg cellulitis. In the past 1 year, the patient continued to take Aescuven forte tablets orally and the left lower extremity continued to be treated with elastic bandage compression.

This study was approved by the ethical committee of the Second Hospital of Shandong University. All procedures carried out on the patient complied with the Helsinki Declaration. Informed consent was obtained from the patient for publication of this case report details.

## 3. Discussion

KTS complicated by frequent cellulitis seriously affects patients’ quality of life. Except for conservative treatment including antimicrobial agents and elastic compression,^[4,5]^ there seems to be no superior treatment. This requires a clear understanding of the pathogenesis of the disease. DUS combined with CTV, which are complementary to each other, can provide an objective basis for exploring the hemodynamic mechanism of KTS. CTV volume imaging can intuitively show the distribution of normal and malformed venous systems in the lower extremities, as well as their interrelation.^[10–12]^ Axial CT images can clearly show the occurrence of lymphedema in the superficial fascia. DUS can provide “live” data for CT static images, including vessel diameter, hemodynamic characteristics, venous reflux time, etc. According to the above methods, the anatomical structure and location of the deep vein system and the great saphenous vein system in the man were normal, and the function of venous return was normal, except for the thin diameter. The malformed vein is 4.6 times the diameter of the superficial femoral vein, and its valve structure is missing. The diameter of the largest penetrating vein is 3.7 times that of the superficial femoral vein. It is speculated that the venous hypertension generated by the malformed venous system continues to act on the leg, destroying the normal venous lymphatic circulation system, leading to lymphatic circulation disorder, and thus producing lymphedema. Unfortunately, persistent venous hypertension in the lower leg leads to skin nutritional disorders that are prone to cellulitis. It was reported that lymphatic venous anastomosis of lower extremity effectively alleviated the clinical symptoms of KTS, which also confirmed our speculation. Therefore, blocking the adverse effects of venous hypertension caused by the malformed venous system on the lower extremities may be the key to solve this disease.

If the deep venous system of the lower extremity is anatomically normal, removal of the malformed venous system does not aggravate extremity swelling.^[6,12]^ On the contrary, it may significantly improve the patient clinical symptoms. The outcome of this man supports this view. It is hypothesized that the malformed venous system plays a major role in venous return from birth, resulting in “a semi-dormant state” of the deep venous system of lower extremity. The successful implementation of CHIVA surgical concept in the treatment of varicose veins of lower extremity provides ideas for the treatment of KT syndrome.^[13]^ Treatment of KTS may require multiple surgeries rather than one. The purpose of the operation is to improve the clinical symptoms and quality of life.^[14]^ In this case, we tried to close the malformed vein and its associated perforating vein in the lower leg, which blocked the recurrence of cellulitis and significantly improved the patient quality of life.

There are many ways to remove malformed veins.^[7–9,15]^ In this case, due to the man lymphedema of the leg, frequent cellulitis, and skin nutritional disorders, any direct puncture or incision may result in non-healing of the incision or ulceration. Therefore, in the first operation, we performed foam sclerotherapy through the right femoral vein approach into the malformed vein of the left lower extremity. Notably, although foam sclerotherapy was effective in controlling the recurrence of cellulitis in the short term, there was a tendency to recanalize the closed malformed veins on CT follow-up at 6 months. Therefore, foam sclerotherapy may not be an optimal treatment option in long-term follow-up, but may provide a safe skin environment for other treatments such as surgical excision.

The major limitation of our work was that, due to the small sample size, the general rule regarding the extent and timing of resection of the malformed vein could not be summarized. Responses to questions such as whether to perform prophylactic resection or therapeutic resection (after the onset of ulceration or infection of the lower extremity), whether to perform one-stage complete resection or a staged segmental resection of the malformed vein, require clinical observation with a large sample size.

## 4. Conclusions

Foam sclerotherapy can significantly improve the clinical symptoms of KTS, such as cellulitis, and provide a safe skin environment for the implementation of other surgical methods, based on the evaluation of the pathological characteristics of KTS by DUS combined with CTV.

## Author contributions

**Conceptualization:** Mengtao Wu.

**Data curation:** Fandong Li, Mengtao Wu, Peng Wu, Dianjun Tang.

**Investigation:** Mengtao Wu, Peng Wu.

**Supervision:** Mengtao Wu.

**Writing – original draft:** Fandong Li, Peng Wu, Dianjun Tang.

**Writing – review & editing:** Mengtao Wu.
